# Gene Expression Profile Reveals Abnormalities of Multiple Signaling Pathways in Mesenchymal Stem Cell Derived from Patients with Systemic Lupus Erythematosus

**DOI:** 10.1155/2012/826182

**Published:** 2012-08-27

**Authors:** Yu Tang, Xiaolei Ma, Huayong Zhang, Zhifeng Gu, Yayi Hou, Gary S. Gilkeson, Liwei Lu, Xiaofeng Zeng, Lingyun Sun

**Affiliations:** ^1^Department of Rheumatology and Immunology, Nanjing Drum Tower Hospital Clinical College of Traditional Chinese and Western Medicine, Nanjing University of Chinese Medicine, 321 Zhongshan Road, Nanjing, Jiangsu 210008, China; ^2^Immunology and Reproductive Biology Lab, Nanjing University Medical School, Nanjing, Jiangsu 210008, China; ^3^Division of Rheumatology, Medical University of South Carolina, Charleston, SC 29403, USA; ^4^Department of Pathology and Center of Infection and Immunology, The University of Hong Kong, Hong Kong; ^5^Department of Rheumatology, Peking Union Medical College Hospital, Peking 100730, China

## Abstract

We aimed to compare bone-marrow-derived mesenchymal stem cells (BMMSCs) between systemic lupus erythematosus (SLE) and normal controls by means of cDNA microarray, immunohistochemistry, immunofluorescence, and immunoblotting. Our results showed there were a total of 1, 905 genes which were differentially expressed by BMMSCs derived from SLE patients, of which, 652 genes were upregulated and 1, 253 were downregulated. Gene ontology (GO) analysis showed that the majority of these genes were related to cell cycle and protein binding. Pathway analysis exhibited that differentially regulated signal pathways involved actin cytoskeleton, focal adhesion, tight junction, and TGF-*β* pathway. The high protein level of BMP-5 and low expression of Id-1 indicated that there might be dysregulation in BMP/TGF-*β* signaling pathway. The expression of Id-1 in SLE BMMSCs was reversely correlated with serum TNF-*α* levels. The protein level of cyclin E decreased in the cell cycling regulation pathway. Moreover, the MAPK signaling pathway was activated in BMMSCs from SLE patients via phosphorylation of ERK1/2 and SAPK/JNK. The actin distribution pattern of BMMSCs from SLE patients was also found disordered. Our results suggested that there were distinguished differences of BMMSCs between SLE patients and normal controls.

## 1. Introduction

Systemic lupus erythematosus (SLE) is a chronic autoimmune disease characterized by multiorgan involvement including renal, cardiovascular, neural, musculoskeletal, and cutaneous systems and remarkable variability in clinical presentation and the etiopathogenesis of SLE remains unclear [1]. In recent years, several studies suggest that SLE may be identified as a stem cell disorder, the etiopathogenesis of this autoimmune disease is attributable to defects in the bone marrow microenvironment, mainly in the hematopoietic stem cells (HSCs) [2], and the bone marrow transplantation (BMT) has a curative effect on systemic autoimmune disease in (NZB × NZW) F1, BXSB, and (NZW × BXSB) F1 mice [3, 4]. 

Stromal cells in bone marrow, also called bone-marrow-derived mesenchymal stem cells (BMMSCs), are one of important components of bone marrow microenvironment, which play a crucial role in the growth, differentiation, and function of HSCs [5]. In addition, BMMSCs can differentiate into a variety of cell types including osteoblasts, chondrocytes, adipocytes, and myoblasts [6–9] and possess immuno-modulatory properties such as inhibiting T-cell proliferation in vitro [10, 11]. Studies on animal models showed BMMSCs from lupus BXSB mice were slower to grow, less proliferative, and harder to differentiate into osteoblasts compared with those from healthy C57/Bl6 mice, and the deficiencies were associated with structural alterations in the gap junction protein Cx43 [12]. BMMSCs from SLE patients have impaired hematopoietic function [13], demonstrating early signs of senescence [14]. In our previous study, we reported BMMSCs derived from SLE patients showed significantly decreased bone-forming capacity and impaired reconstruction of bone marrow osteoblastic niche in vivo [15]. Moreover, the mRNA level of IL-6 and IL-7 were downregulated in BMMSCs from SLE patients [16]. So we hypothesize that SLE might not only be a stem cell disease, but also a BMMSCs disorder. Based on this hypothesis, in the clinical setting, we intravenously infused allogenic BMMSC or umbilical cord mesenchymal stem cell (UCMSC) to SLE patients, the majority of recipients experienced rapid improvement postinfusion [15, 17–19]. Those studies indicated that, as one of components in the bone marrow microenvironment, BMMSCs dysfunction probably partook in the pathogenesis of SLE and correction of the abnormalities might contribute to the disorder improvement. 

Nonetheless, relatively little is known about the cellular and molecular mechanisms underlying the control of mesenchymal stem cell (MSC) proliferation, differentiation, and survival. Recent results have demonstrated multiple signaling pathways involved in the functions of MSCs. For example, the osteogenic differentiation of MSCs induced by bone morphogenetic proteins-2 (BMP-2) may be mediated by coordinated activation of Notch, Wnt, and transforming growth factor-*β* (TGF-*β*) signaling pathways [20]; MSCs were activated by TLR ligands leading to modulation of the differentiation, migration, proliferation, survival, and immunosuppression capacities [21–23]. But the studies concerning pathways involved in the deficiency of BMMSCs from SLE patients are almost blank. 

In this study, using the microarray assay, we firstly found that there were significant differences in gene expression profile (GEP) of BMMSCs between SLE patients and normal controls. And in the further investigation, we confirmed that there were abnormalities in actin cytoskeleton, cell cycling regulation, BMP/TGF-*β*, and MAPK signaling pathways in BMMSCs from SLE patients.

## 2. Materials and Methods

### 2.1. Patients and Controls

Bone marrow (BM) was obtained for cDNA microarray from 4 SLE patients according to the American College of Rheumatology criteria [24]. All were female, and the mean age was 37 ± 11 years (range 20~44). The demographic data and clinical features of SLE patients were listed in Table 1. The normal controls were 1 male and 3 females, with a mean age of 39 ± 7 years (range 29~45). Further qRT-PCR was performed from 10 female patients (mean 40 ± 14 years, range 15~60 years) and 10 female normal controls (mean 41 ± 14 years, range 24~65 years). All SLE patients had active disease with a SELENA-SLEDAI (Systemic Lupus Erythematosus Disease Activity Index) [25] score of more than 10 at the time of bone marrow aspiration. All participants gave written consent to the study which was approved by the Ethics Committee of the Affiliated Drum Tower Hospital of Nanjing University Medical School. 

### 2.2. Cell Culture and Flow Cytometry

BM was taken from the iliac crest of SLE patients and normal controls, resuspended by phosphate-buffered saline (PBS), and then layered over 1.077 g/mL Ficoll (TBD, Tianjin, China) solution before being centrifuged at 600 ×g for 20 minutes at room temperature. The mononuclear cells were collected and resuspended in low glucose Dulbecco Modified Eagle Medium (L-DMEM, Gibco) supplemented with 10% heat inactivated fetal bovine serum (FBS, Invitrogen, USA) and 1% antibiotic-antimycotic solution and plated at a density of 2 × 10^7^ cells per 25 cm-dish. The cultures were maintained at 37°C in a 5% CO_2_ incubator, and the medium was changed after 48 hours and then every three days. When the MSCs were confluent, the cells were recovered by the addition of 0.25% trypsin-EDTA (Gibcoth) and then replated at a density of 1 × 10^6^ cells per 25 cm dish. Cells at passage 3 were consequently analyzed by flow cytometry as described previously [16]. 

### 2.3. Microarray Hybridization

BMMSCs were placed in Trizol (Invitrogen, USA) and processed for RNA extraction using the RNeasy kit according to the instructions of the manufacture (Qiagen, Valencia, CA). The universal human reference RNA samples which comprised of 10 different cell lines of humans (Stratagene Corporation, USA) were used as a common reference in the two channel microarray. Total RNA was reverse transcribed, and the cDNA of BMMSCs from SLE patients and normal controls was added with Cy3-dCTP while the cDNA of human reference was added with Cy5-dCTP in the present with Klenow enzyme (GE Healthcare Cat. Nos. PA 55021/PA 53021) [26]. Microarray analysis was performed in CapitalBio Corp (Beijing, China) using 22 K Human Genome Array. The slide contains gene-specific 70-mer oligonucleotides representing 21, 329 human genes including four human housekeeping genes as positive controls and twelve random negative controls that are designed to have no significant homology with known human DNA sequences as negative controls. Labeled samples were quantitatively adjusted based on the efficiency of Cy-dye incorporation and mixed into 80 *μ*L hybridization solution (3 × SSC, 0.2% SDS, 25% formamide, and 5 × Denhart's). DNA in hybridization solution was denatured at 95°C for 3 min prior loading on a microarray. The array was hybridized at 42°C overnight and washed with two consecutive washing solutions (0.2% SDS, 2 × SSC at 42°C for 5 min, and 0.2% SSC) for 5 min at room temperature. Finally, arrays were scanned with a confocal LuxScan 10 KA scanner (CapitalBio). The data of obtained images were extracted with LuxScan 3.0 software (CapitalBio). Genes with the signal intensity more than 800 (Cy3 or Cy5) were regarded as the expressed ones. In every two channel slides, the intensity ratio of the Cy3 to Cy5 of each spot was calculated after normalization with LOWESS regression. Statistical data and differential analysis files were generated by using SAM software 3.0 (Stanford University, Stanford, CA, USA). The significant changed genes were selected based on *P* value < 0.05 and >2-fold as criteria. All the differentially expressed genes were analyzed using a free web-based Molecular Annotation System 2.0 (MAS 2.0, http://bioinfo.capitalbio.com/mas3/) [27, 28]. 

All data is MIAME compliant and that the raw data has been deposited in a MIAME compliant database (GEO). The raw data can be seen http://www.ncbi.nlm.nih.gov/geo/query/acc.cgi?acc=GSE21649. The accession number is GSE 21649.

### 2.4. Quantitative Reverse Transcription-Polymerase Chain Reaction

Gene expressions were examined by real time RT-PCR performed by ABI 7500 FAST real-time PCR detection system (Applied Biosystems, USA) using SYBR Green detection mix (TaKaRa, Japan) [16]. The expressions of Id (inhibitor of differentiation or inhibitor of DNA binding)-1, Id-2, Id-3, cyclin D, and cyclin E2 were analyzed from 5~10 samples from other SLE patients and normal controls. The following primers were used in this study:

GAPDH (sense): 5′-TGACTTCAACAGCGACACCCA-3′
(antisense): 5′-CACCCTGTTGCTGTAGCCAAA-3′;
Id-1 (sense): 5′-ACGACATGAACGGCTGTTACTCAC-3′
(antisense): 5′-CTCCAACTGAAGGTCCCTGATGTAG-3′;
Id-2 (sense): 5′-TGTCAGCCTGCATCACCAGA-3′
(antisense): 5′-CCACACAGTGCTTTGCTGTCA-3′;
Id-3 (sense): 5′-TCAGCTTAGCCAGGTGGAAATC -3′
(antisense): 5′-GGCTGTCTGGATGGGAAGGT-3′;
Cyclin D: (sense) 5′-TGATGCTGGGCACTTCATCTG-3′
(antisense): 5′-TCCAATCATCCCGAATGAGAGTC-3′;
Cyclin E2 (sense): 5′-GCCGTTTACAAGCTAAGCAGCAG-3′
(antisense): 5′-CCAGATAATACAGGTGGCCAACAA-3′.

### 2.5. Immunofluorescence Staining

Cells were washed three times with PBS, fixed for 10 min 3.7% formaldehyde in PBS, and permeabilized for 5 min with 0.2% triton X-100 3.7% formaldehyde. The fixed cells were rehydrated with Tris buffered saline (TBS) and incubated for 1 h in blocking solution (3% BSA in TBS), then they were incubated with Alexa Flour 594 conjucted phalloidin (Invitrogen, USA) or phalloidin-FITC (Sigma, USA) antibodies for 1 h at 37°C. Nuclei were counterstained with DAPI (4,6-diamidino-2-phenylindole). Finally, cells were rinsed in TBS, mounted in DABCO/mowiol. Images were acquired using a TCS SP2 confocal microscope (Leica, Germany) or fluorescence microscope (Olympus, Japan).

### 2.6. Immunocytochemistry Staining

Cells were seeded on poly-L-lysine-coated 6-well chamber slides (BD, Bioscience), cultivated for another 3 days. Samples were then fixed with cold acetone for ten minutes followed by incubation in 3% hydrogen peroxide to block the endogenous peroxide activity. To prevent nonspecific antibody binding, slides were preincubated for 30 min in normal goat serum. Slides were then incubated with primary monoclonal antibody against human BMP-5 (Bioword Technology, USA) at 37°C for 1 h, followed by incubated with second antibody MaxVision kit (Maxim Inc., China) for 15 min at room temperature. After a 15-min wash, slides were treated with 3,3′-diaminobenzidine (DAB) for 5 min and finally counterstained with hematoxylin. 

### 2.7. Immunoblotting

Cells were lysed with sodium dodecyl sulfate- (SDS-) sample buffer containing 0.1 M Tris-HCl, 4% SDS, 0.2% Bromophenol Blue, and 5%  *β*-mercaptoethanol. Cell lysates were separated by SDS-PAGE and transferred to a polyvinylidene difluoride (PVDF) membrane (Millipore, Bedford, MA). Blots were probed by anti-phospho- and anti-total-extracellular signal-regulated kinase (ERK)1/2 MAPK antibodies (Cell Signaling Technology Inc.), anti-phospho- and anti-total-P38 MAPK antibodies (Cell Signaling Technology Inc.), anti-phospho- and anti-total-stress-activated protein kinase/c-Jun NH2-terminal kinase (SAPK/JNK) MAPK antibody (Cell Signaling Technology Inc.), anti-cyclin D antibodies (Epitomics Inc.), and anti-cyclinE (Epitomics Inc.) before visualizing with HRP-conjugated secondary antibodies followed by development with FluorChem FC2 System (Alpha Innotech Corporation, USA).

### 2.8. ELISA Analysis

Serum from 10 SLE patients and 20 normal controls were collected, and the concentrations of tumor necrosis factor-*α* (TNF-*α*) of each individual were measured using commercial ELISA kit (R&D) according to the manufactory introduction.

### 2.9. Statistical Analysis

Statistical analyses were performed using SPSS for 16.0. All data were expressed as mean ± SEM. The relative expression of the target genes in SLE samples as compared with that in normal controls was examined using 2^−ΔΔCt^ method [29]. Briefly, for each sample, a value for the cycle threshold (Ct) was determined, defined as the mean cycle at which the fluorescence curve reached an arbitrary threshold. The ΔCt for each sample was then calculated according to the formula Ct target gene-Ct GAPDH; ΔΔCt values were then obtained by subtracting the ΔCt of a reference sample (average ΔCt of the control group) from the ΔCt of the studied samples. Finally, the levels of expression of the target genes in the studied samples as compared with the reference sample were calculated as 2^−ΔΔCt^. A *P* value of 0.05 or less (*P* < 0.05) by independent Student's *t* test or nonparametric test was considered statistically significant.

## 3. Results

### 3.1. Unsupervised Hierarchical Clustering in the BMMSCs from SLE Patients and Normal Controls

**Figure 1:**
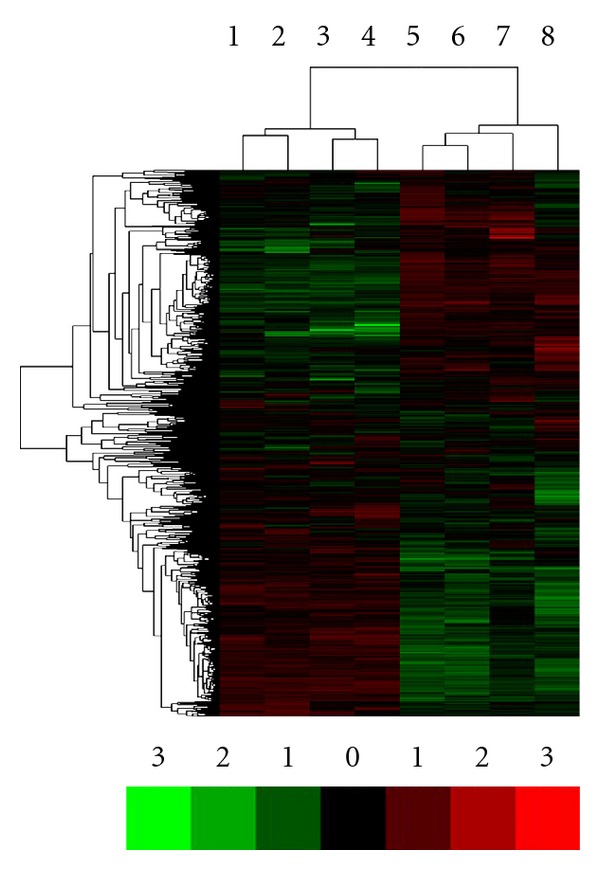
Genes differentially expressed in BMMSCs between SLE patients and normal controls. Genes were shown by the ratio of hybridization intensity between normal control and SLE BMMSCs. The ratio ≥2 or ≤0.5 was considered significant. Genes highly expressed in BMMSCs from normal controls were highlighted in green, while those highly expressed in BMMSCs from SLE patients were highlighted in red.

Firstly, we sought to investigate whether the gene expression profiles were globally different between BMMSCs from patients with SLE and normal controls. We used the total number of 8, 769 genes detected to perform the unsupervised hierarchical clustering after faint spots were removed. As expected, hierarchical clustering of the 8 BMMSCs samples fell into 2 groups displaying different expression patterns of those 8, 769 genes. One cluster consisted of samples from SLE patients (samples 1~4) and the other cluster consisted of those from normal controls (samples 5~8, Figure 1). These data suggested the existence of different gene expression patterns of BMMSCs between SLE patients and normal controls. 

### 3.2. Gene Ontology (GO) and Pathway Analysis of BMMSC

**Figure 2:**
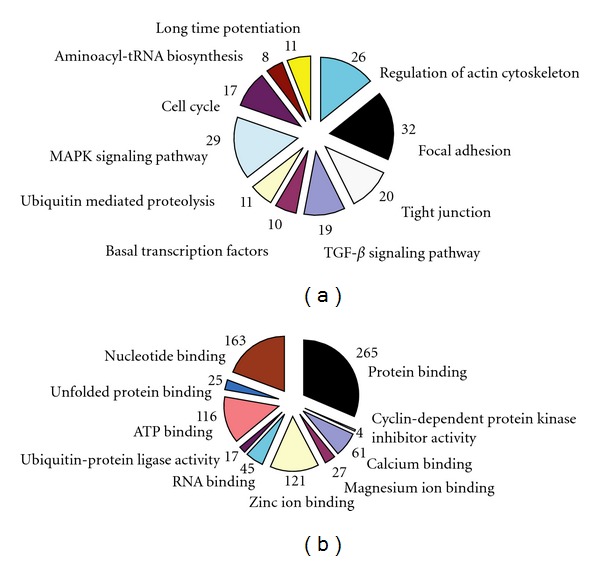
Classification of the differentially expressed genes according to the pathway and GO analysis by MAS software. (a) The top 10 statistically significant (*P* < 0.001) pathways. (b) The top 10 statistically significant (*P* < 0.001) molecular functions by GO analysis. The numbers indicated the differentially expressed genes in the specific pathway or function.

There were 1, 905 genes found to be differently expressed between the SLE patients and the normal controls using SAM software combined *P*-value < 0.05 and >2-fold criteria. Of those genes, 652 were upregulated in the BMMSCs of SLE patients, while other 1, 253 were downregulated. The functions of differentially expressed pathways included actin cytoskeleton, focal adhesion, TGF-*β* signaling, and tight junction. The altered expression of 26 genes was found to be involved in regulating actin cytoskeleton pathway, among which 6 genes were up-regulated while 20 down-regulated in BMMSCs from SLE patients. Interestingly, most genes in TGF-*β* signaling pathway were downregulated except for BMP5. Moreover, GO analysis found that genes involved in the control of the cell cycle, protein binding, and calcium ion binding showed the most significant differences among gene expression profiles (all *P* < 0.0001; Figure 2). The differentially expressed genes in regulation of actin cytoskeleton and TGF-*β* signaling were listed in Table 2. The expressions of SMAD1, BMPR1A, ACTB, and ARPC5 by microarray assay were confirmed by qRT-PCR analysis. The four selected genes were initially validated by qRT-PCR in the RNA samples used for the microarrays. As expected, the qRT-PCR data showed significant differences between SLE patients and normal controls and confirmed the direction of the fold changes (supplementary Figure  1; see Supplementary material available online at doi:10.1155/2012/826182).

### 3.3. Abnormal Actin Cytoskeleton Distribution Pattern in BMMSCs from SLE Patients

**Figure 3:**
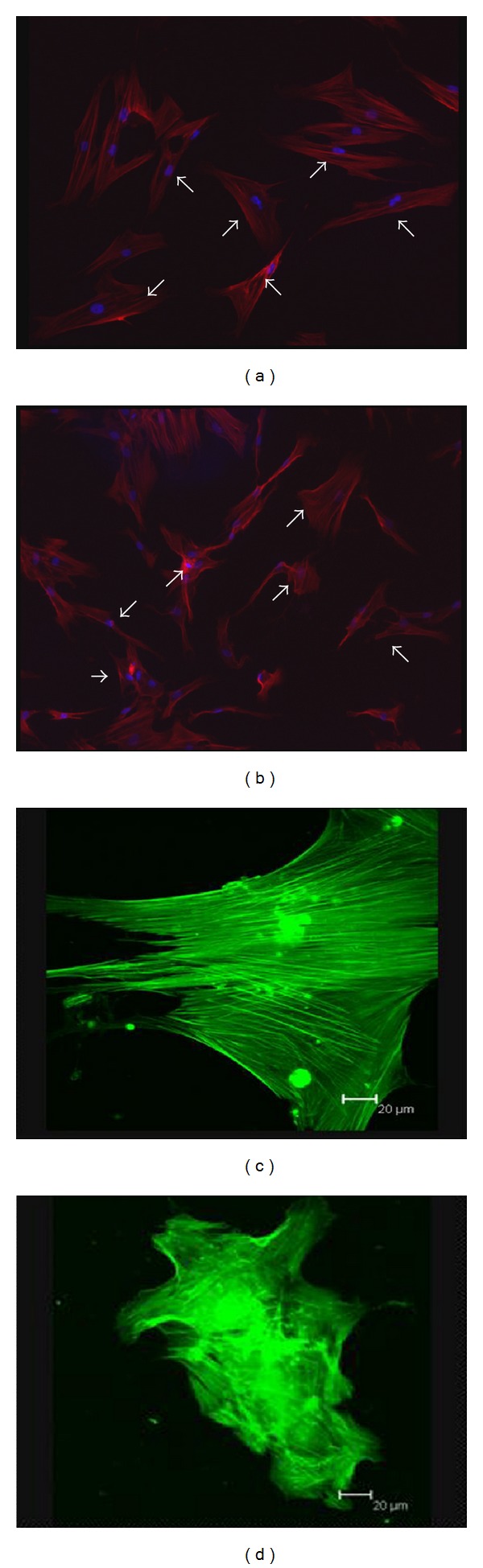
Actin distribution patterns in BMMSCs from SLE patients or the normal controls. (a, b) Actin filaments were stained with Alexa Flour 594 conjucted phalloidin (red) and nuclei were counterstained with DAPI (blue). Cells (white arrows) were observed by fluorescence microscope. (a): BMMSCs from normal controls, (b): BMMSCs from SLE patients. (magnification ×200) (c, d) Actin filaments was stained with phalloidin-FITC and observed under confocal microscopy. (c): BMMSC from one normal control, (d): BMMSC from one SLE patient (Bar = 20).

Consistent with our previous findings, flow cytometric analysis showed CD29, CD44, and CD105 expression of >95%, in parallel with CD45, CD34, CD14, and HLA-DR expression of <5%  (supplementary Figure 2). Although BMMSCs from SLE patients and normal controls showed similarly fibroblast-like morphology as observed by light microscopy [16], the actin distribution pattern in BMMSCs from SLE patients, distinct from that from normal controls (Figure 3(a)), exhibited an irregular and twisted pattern under fluorescence microscope (Figure 3(b)). Under confocal microscopy, BMMSCs from normal controls displayed a pattern of parallel actin stress fibers extending across the entire cytoplasm as revealed by phalloidin staining (Figure 3(c)), while F-actin in BMMSCs from SLE patients was disorganized and condensed on the edge of cytoplasm (Figure 3(d)). 

### 3.4. Altered Protein Expression in Regulating Cell Cycle

**Figure 4:**
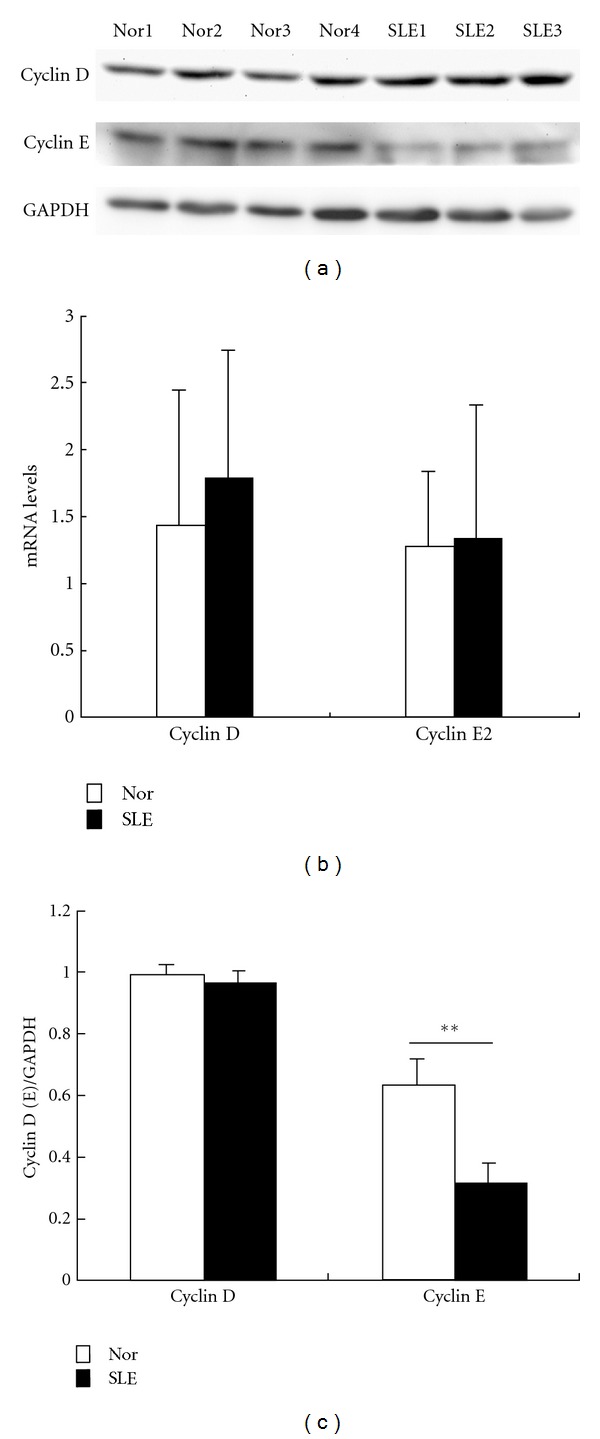
mRNA and protein levels of cyclin D and cyclin E in BMMSCs from SLE patients. (a) Immunoblotting analysis of cyclin D and cyclin E. (b) qRT-PCR studies of the expression of cyclin D and cyclin E2 in BMMSCs from SLE and normal controls (*n* = 5, *P* > 0.05). Results were shown as mean ± SEM, each performed with triplicate samples. (c) Quantity analysis showed low protein level of cyclin E in cells from SLE patients, **P < 0.01 by Student's *t*-test. SLE: systemic lupus erythematosus, Nor: normal controls.

Since microarray analysis showed altered expression profile of genes involved in cell cycle, we evaluated the mRNA and protein levels of cyclin D and cyclin E in samples from 5 SLE patients. No difference was found in the levels of cyclin D and cyclin E2 transcripts between BMMSCs from SLE patients and normal controls. However, immunoblotting analysis further revealed reduced protein level of cyclin E in BMMSCs from SLE patients (*n* = 3, *P* = 0.003) (Figure 4). 

### 3.5. Abnormal Gene and Protein Expressions in BMP/TGF-*β* Signaling Pathway

**Figure 5:**
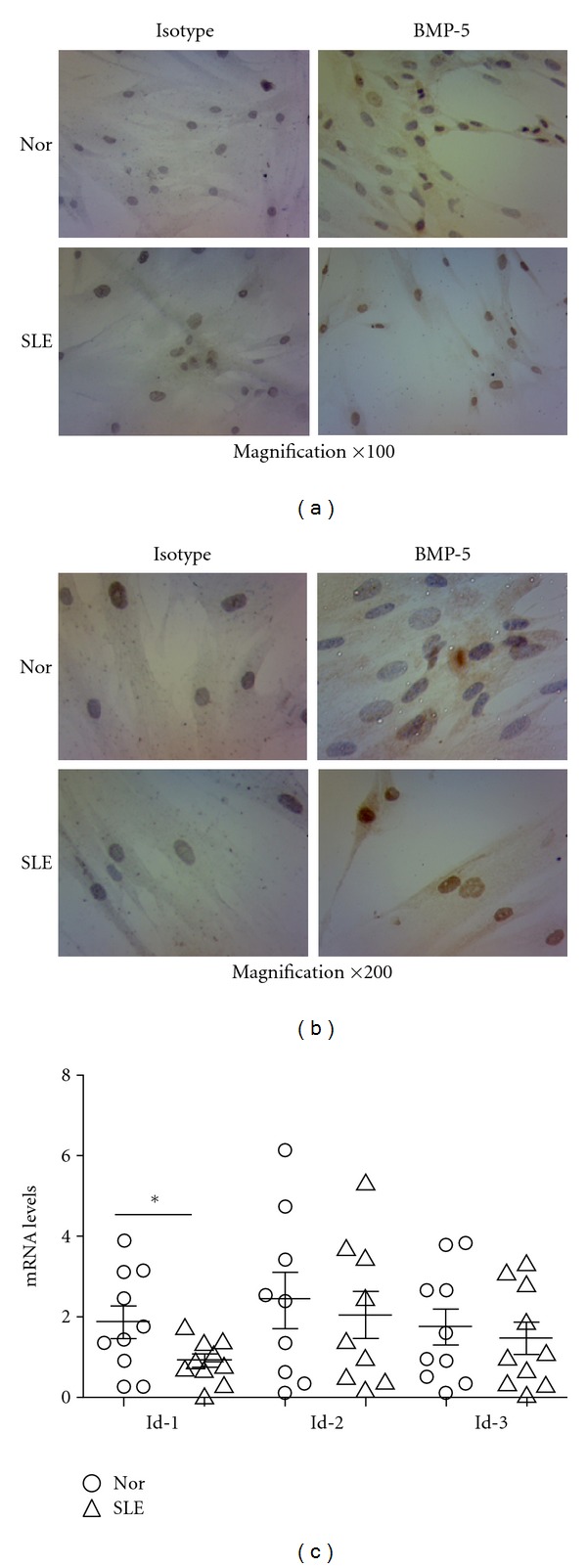
Immunohistochemical detection of BMP-5 protein and mRNA levels of Id-1, Id-2, Id-3 in BMMSCs. (a) magnification ×100, (b) magnification ×200. The left pictures are isotype controls, the right are BMP-5 immunohistochemical staining. (c) qRT-PCR studies of the expression of Id-1, Id-2, and Id-3 in BMMSCs from SLE patients and normal controls (*n* = 10). *P < 0.05 by nonparametric test of SPSS 16.0 software. SLE: systemic lupus erythematosus, Nor: normal controls.

In addition, we performed immunostaining experiments to detect the protein level of BMP-5, which was the only upregulated gene in BMP/TGF-*β* signaling pathway in microarray. Moreover, the expressions of target gene of BMP signaling pathway, including Id-1, Id-2, and Id-3, from 10 samples of SLE patients and normal controls were analyzed by qRT-PCR. Most of BMMSCs from both normal controls and SLE patients were positively stained cells. However, BMMSCs from normal controls showed light brown staining in cytoplasm while BMMSCs from SLE patients were dark brown stained in both nuclei and cytoplasma, suggesting that BMP-5 protein expression was upregulated in BMMSCs from SLE patients. Among the target genes, only the expression of Id-1 was lower in SLE (0.89 ± 0.51) compared with normal controls (1.86 ± 1.26) (*n* = 10, *P* = 0.037). The results indicated that the BMP signaling pathway appeared to be dysregulated in BMMSCs from SLE patients (Figure 5).

### 3.6. Activated MAPK Pathway in BMMSCs from SLE Patients

**Figure 6:**
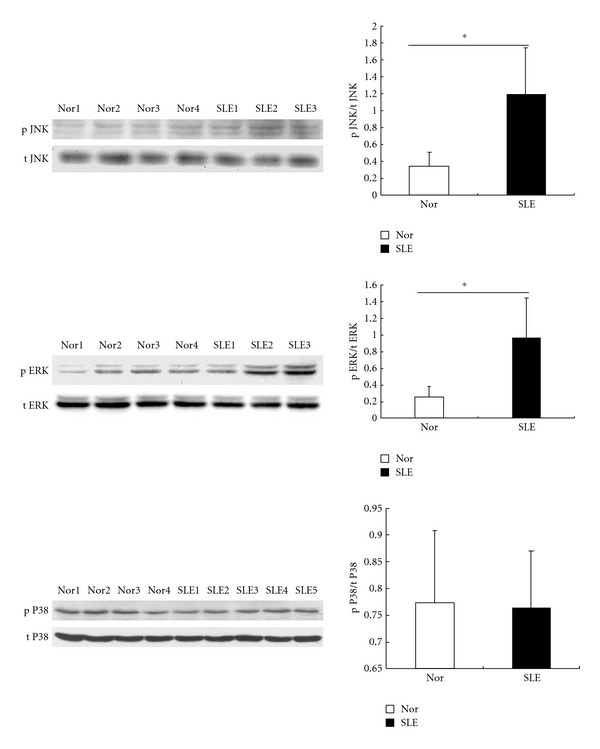
MAPK pathway in BMMSCs from SLE patients. Immunoblotting showed the phosphorylation of JNK and ERK1/2 was higher in BMMSCs from SLE patients. Results were shown as mean ± SEM. *P < 0.05 (SLE versus normal controls by Student's *t*-test). SLE: systemic lupus erythematosus, Nor: normal controls.

Another cascade that appeared to be disordered was the MAPK pathway. As shown in Figure 6, the phosphorylation of ERK1/2 (*n* = 3, *P* = 0.03) and SAPK/JNK (*n* = 3, *P* = 0.03) were higher in BMMSCs from SLE patients, as compared with normal controls (*n* = 4), while the phosphorylation of P38 showed similar levels in BMMSCs between SLE patients and normal controls, suggesting a partially activated MAPK pathway in BMMSCs from SLE patients.

### 3.7. Id-1 Associated with Serum TNF-*α* Level in SLE Patients

**Figure 7:**
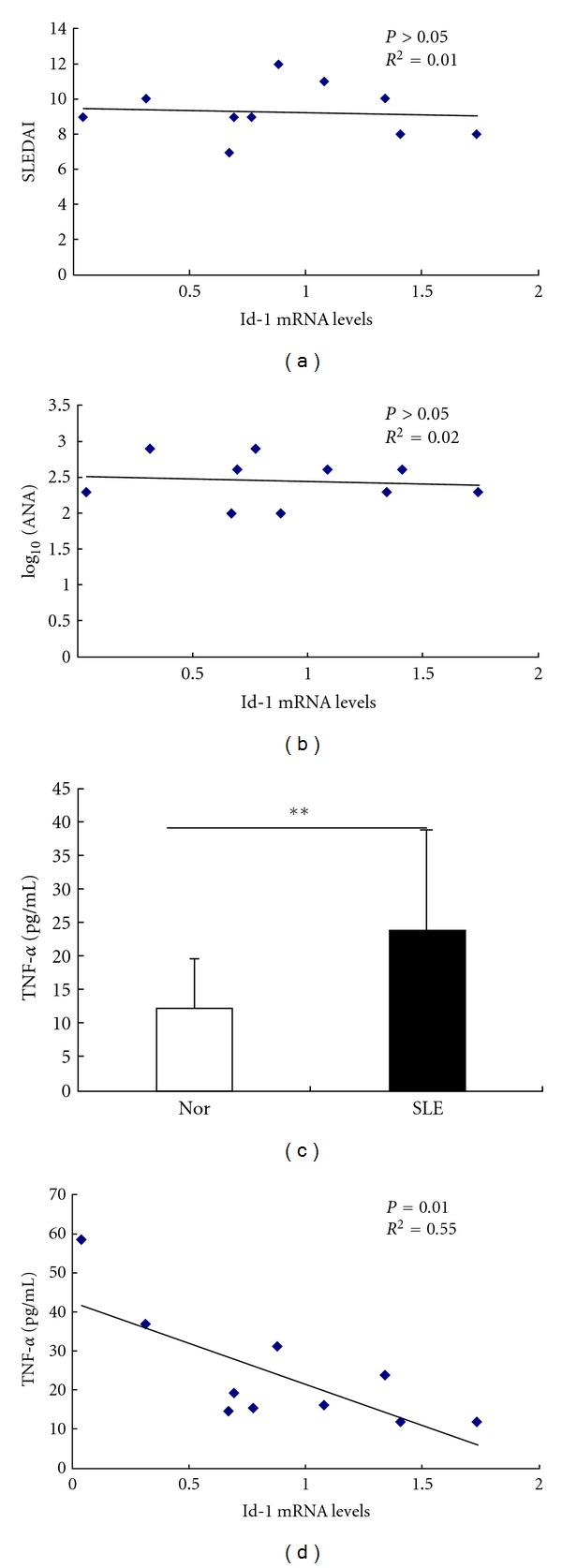
The correlation of Id-1 mRNA expression with serum TNF-*α* levels in SLE patients. (a, b) Id-1 mRNA level was not correlated with antinuclear antibodies (ANA) levels and SLE disease activity index (SLEDAI) score of SLE patients. (c) The serum level of TNF-*α* in SLE patients (*n* = 10) were higher than that of normal controls (*n* = 20). **P < 0.01 versus normal controls by Student's *t*-test. (d) Id-1 mRNA expression was reversely correlated with serum TNF-*α* level of SLE patients (*P* = 0.01, *r* ^2^ = 0.55). SLE: systemic lupus erythematosus, Nor: normal controls.

In order to identify the relationship between the differentially expressed genes and the clinical outcome, correlation analysis was used between Id-1 mRNA levels and serum levels of antinuclear antibodies (ANAs), TNF-*α*, and SLEDAI in SLE patients. The level of TNF-*α* in the serum of SLE patients (*n* = 10) was higher than that in normal controls (*n* = 20) (*P* = 0.006). Id-1 mRNA levels had no correlation with ANA, SLEDAI, but it was reversely correlated with serum level of TNF-*α* in SLE patients (Figure 7). In addition, Id-1 mRNA levels of BMMSCs from normal controls had no correlation with their serum levels of TNF-*α* (see supplementary Figure 3,  *n* = 10, *P* = 0.76).

## 4. Discussion

Previous studies using microarray in SLE examined gene expression in peripheral blood mononuclear cells (PBMCs) and showed interferon- (IFN-) inducible and granulopoiesis signatures correlating with both disease severity and disease activity [30, 31]. IFN-related genes and genes involved in extra-cellular matrix (ECM) homeostasis were also found differentially expressed in target organs, such as lupus glomeruli and synovium of SLE patients in some studies [29, 32]. One study differentiated active SLE from inactive by the microarray analysis of the bone marrow mononuclear cells (BMMCs), and the upregulated genes in SLE patients were involved in cell death and granulopoiesis [33]. In our study, genes in regulation of cell cycle, actin cytoskeleton regulation, TGF-*β*, focal adhesion, and MAPK pathways, rather than type I interferon signature were found to be differentially expressed in the BMMSCs from SLE patients, suggesting the distinct role of bone marrow, especially the stromal cells in regulating the immune response.

Although the morphological characteristics of BMMSCs from SLE patients was the same as the normal controls [16], we observed under confocal and fluorescence microscopes in this study that the F-actin of BMMSCs from SLE patients was confused and condensed on the edge of cytoplasm, which was absolutely different from normal controls. This actin distribution of BMMSCs from SLE patients supported the notion that MSCs from SLE patients tended to be senescent [8]. Actin filaments form the cytoskeleton with microtubules and their prokaryotic cousins play central roles in cell shape, motility, and chromosome segregation control [34, 35]. Moreover, recent studies found actin filaments were closely related to the apoptosis, aging, and malignant transformation of cells [36, 37]. Those studies in combination with present results indicated that BMMSCs from SLE patients might be abnormal in such functions as migration and aging, which attributes to the disordered actin cytoskeleton.

According to the gene ontology analysis, the most differentially expressed genes were those involved in regulating the cell cycle, which is consistent with the reports that BMMSCs from SLE patients showed lower proliferative capacity compared with normal controls [14, 16]. In this study, we examined the mRNA and protein levels of only two members of cyclin family, cyclin D and cyclin E2. Although no difference was found in the mRNA level in BMMSCs between SLE patients and normal control, immunoblotting analysis demonstrated that the cyclin E expression was lower in the BMMCs from SLE patients, suggesting a deficiency at protein level. This deficiency in regulation of cell cycle might result in the decreased cell proliferative capacity of BMMSCs in SLE patients.

BMPs are multifunctional growth factors that belong to the TGF-*β* superfamily. Studies have shown that BMP signaling plays critical roles in bone formation and cartilage development [38]. Specific BMPs such as BMP-2, BMP-6, and BMP-9 promote the differentiation of MSCs into osteoblasts in vitro [39]. The BMP signaling cascade initiate from the binding with BMP receptors. Binding of an extracellular ligand promotes the dimerization of the two serine/threonine protein kinases. The type-II kinase phosphorylates the type-I receptor. Activation of the type-I receptor initiates phosphorylation of downstream effector proteins, such as receptor-regulated Smads (R-Smads), including Smad-1, 2, 3, 5, and 8, leading to signal transduction. Following activation, the R-Smad protein forms a heterooligomeric complex with a common mediator Smad (Co-Smad; Smad4), which translocates into the nucleus and regulates the transcription of target genes, such as Rux2, Msx2, and osterix [40]. According to the results of the microarray, most of genes in the BMP pathway were decreased including Smad-1, Smad-5, BMPR1A and the target gene Id-1. As the phosphorylation process controls the activity of Smad-1, Smad-5, and BMPR1A, we only confirmed the mRNA level of some of the target genes and the protein level of BMP-5. 

Id genes are thought to be the most targeted genes by BMP-Smad signaling. Four Id proteins (Id-1 to -4) have been identified in mammals, which are critical in controlling the differentiation and proliferation of myeloid lineages [41]. Previous studies indicated that Id-3 was overexpressed in SLE peripheral blood cells [42] and Id-1 transcription was upregulated by IL-6 stimulation in the B6.Sle1.Yaa mice model [43]. When compared with normal controls, we found that the Id-1 mRNA level was lower in BMMSCs derived from SLE patients, however, its level was not correlated with ANA level or SLADAI score of SLE patients. Similar to the results described by others [44, 45], TNF-*α* level was also found higher in the serum of SLE patients in our study, which was reversely correlated with Id-1 mRNA level. This result further confirmed those of microarray and suggested there might be a dysregulated BMP pathway in BMMSCs from SLE patients, which possibly contribute to the osteogenesis impairment and osteoblastic niche deficiency in MRL/lpr mice and SLE patients [15]. 

MAPK cascade is an important pathway that transmits extracellular signals into cytoplasma to initiate cellular processes such as proliferation, differentiation, and development. The three well-characterized subfamilies of MAPKs include the extracellular signal-regulated kinases (ERK1/2), c-Jun NH2-terminal kinases (JNK-1/2/3), p38. Many growth factors can trigger MAPK pathway including epidermal growth factor (EGF), fibroblast growth factor (FGF), and platelet-derived growth factor (PDGF). In addition, chemokines such as SDF-1 could stimulate human MSCs migration through increased phosphorylation of ERK [46], and Wnt3a could induce a rapid and transient activation of MAPKs p38 and ERK1/2 leading to increased alkaline phosphatase activity and nodule mineralization in murine C3H10T1/2 mesenchymal cells [47]. Moreover, many inflammatory cytokines, such as IL-1 and TNF-*α*, could inhibit the osteoblastic differentiation via phosphorylation of ERK1/2 and SAPK/JNK [48, 49]. We found in this study an increased phosphorylation of ERK1/2 and SAPK/JNK in BMMSCs from SLE patients and postulate that this activation might result from the inflammatory environment in the bone marrow of SLE patients. 

## 5. Conclusion

In conclusion, our present study revealed absolutely different gene profile pattern of BMMSCs from SLE patients and showed disordered actin cytoskeleton in BMMSCs from SLE patients. Furthermore, we found abnormalities in cell cycling regulation, BMP/TGF-*β* and MAPK pathways. Our findings suggest BMMSCs, as a component of bone marrow, may play an important role in the etiopathogenesis of SLE.

## Supplementary Material


**Supplementary Figure 1: Verification of data from microarray hybridization by quantitative Real-time PCR. (A)** The mRNA levels of SMAD1, BMPR1A, ACTB and ARPC5 in BMMSCs from SLE patients and normal controls by qRT-PCR. Results were shown as mean ± SEM, each performed with triplicate samples. ∗P<0.05 by Student's t-test (n = 4). **(B)** The ratio of SLE to normal controls. The folds of SLE to normal controls of Smad1, BMPR1, ARPC5, ACTB were 2.16-, 2.52-, 1.89- and 2.12 respectively by qRT-PCR. The intensity folds of normal to SLE BMMSCs of selected genes between microarray and qRT-PCR were assessed using the independent Student's t-test. There was no statistical significance in the ratios between the methods of microarray and qRT-PCR (n = 4, P>0.05). SLE: systemic lupus erythematosus, Nor: normal controls.
**Supplementary Figure 2:** Flow cytometry analysis showed that the BMMSCs from SLE patients at passage three were CD29, CD44 and CD105 positive, while CD14, CD34, CD45, and HLA-DR negative cells. The shadow showed negative controls. SLE: systemic lupus erythematosus, Nor: normal controls.
**Supplementary Figure 3: The correlation of Id1 with TNF-*α* in normal controls**. Id-1 mRNA levels of BMMSCs from normal controls had no correlation with their serum levels of TNF-*α* (n = 10, *p* = 0.76).

## Figures and Tables

**Table 1:** Demographic data and clinical features of SLE patients for cDNA microarray analysis.

Patient no.	Sex/Age (yrs)	Disease duration (months)	SLEDAI	Clinical manifestations	Therapy
1	F/20	84	14	Nephritis, arthralgia, vasculitis	Pred, HCQ, CYC
2	F/44	12	10	Nephritis, arthralgia, cytopenia	Pred, HCQ, CYC
3	F/43	240	19	Nephritis, cytopenia, interstitial pneumonia	Pred, HCQ, CYC
4	F/42	6	20	Nephritis, cytopenia, interstitial pneumonia, polyserositis	Pred, HCQ, CYC

Pred: Prednisone, CYC: Cyclophosphamide, HCQ: Hydroxychloroquine.

**Table 2:** Differentially expressed genes in regulation of actin cytoskeleton and TGF-*β* signaling between SLE patients and normal controls.

Upregulated genes in actin cytoskeleton pathway	Fold	*P* value
ACTN4	2.30	≤1.0*E* − 6
ACTB	2.41	≤1.0*E* − 6
VAV1	4.08	≤1.0*E* − 6
MATK	2.85	≤1.0*E* − 6
ITGB5	8.46	≤1.0*E* − 6
ITGB4	2.43	≤1.0*E* − 6

Downregulated genes in actin cytoskeleton pathway		

KRAS	5.62	≤1.0*E* − 6
ARPC3	2.17	≤1.0*E* − 6
ARPC4	2.10	≤1.0*E* − 6
ARPC5	2.53	≤1.0*E* − 6
NRAS	2.01	≤1.0*E* − 6
GNG12	2.17	≤1.0*E* − 6
NCKAP1	3.16	≤1.0*E* − 6
ITGA1	3.63	≤1.0*E* − 6
CRKL	2.71	≤1.0*E* − 6
ITGB5	2.58	≤1.0*E* − 6
PPP1CC	3.36	≤1.0*E* − 6
CFL2	2.06	≤1.0*E* − 6
ROCK2	2.38	≤1.0*E* − 6
PDGFRA	2.77	≤1.0*E* − 6
F2R	2.57	≤1.0*E* − 6
RDX	2.23	≤1.0*E* − 6
PPP1R12A	2.99	≤1.0*E* − 6
ARHGEF6	2.31	≤1.0*E* − 6
ITGAV	7.84	≤1.0*E* − 6
CRK	2.71	≤1.0*E* − 6

Upregulated genes TGF-*β* signaling		

BMP5	4.23	≤1.0*E* − 6

Downregulated genes TGF-*β* signaling		

SMAD1	2.08	≤1.0*E* − 6
SMAD5	2.60	≤1.0*E* − 6
SMURF2	2.66	≤1.0*E* − 6
ID1	3.81	≤1.0*E* − 6
BMPR1A	3.03	≤1.0*E* − 6
TGFBR1	4.50	≤1.0*E* − 6
TGFBR2	2.01	≤1.0*E* − 6
ACVR1	3.12	≤1.0*E* − 6
CREBBP	2.06	≤1.0*E* − 6
ROCK2	2.38	≤1.0*E* − 6
RPS6KB1	2.64	≤1.0*E* − 6
CDKN2B	2.03	≤1.0*E* − 6
THBS1	3.14	≤1.0*E* − 6
THBS3	2.11	≤1.0*E* − 6
THBS2	3.05	≤1.0*E* − 6
LTBP1	2.27	≤1.0*E* − 6
COMP	4.89	≤1.0*E* − 6
FST	2.23	≤1.0*E* − 6
