# Supplementary figures and images for: Gene Expression Profile Reveals Abnormalities of Multiple Signaling Pathways in Mesenchymal Stem Cell Derived from Patients with Systemic Lupus Erythematosus
Source: Clin Dev Immunol. 2012 Aug 27;2012:826182. doi: 10.1155/2012/826182 (PMC3433142; doi:10.1155/2012/826182)

A

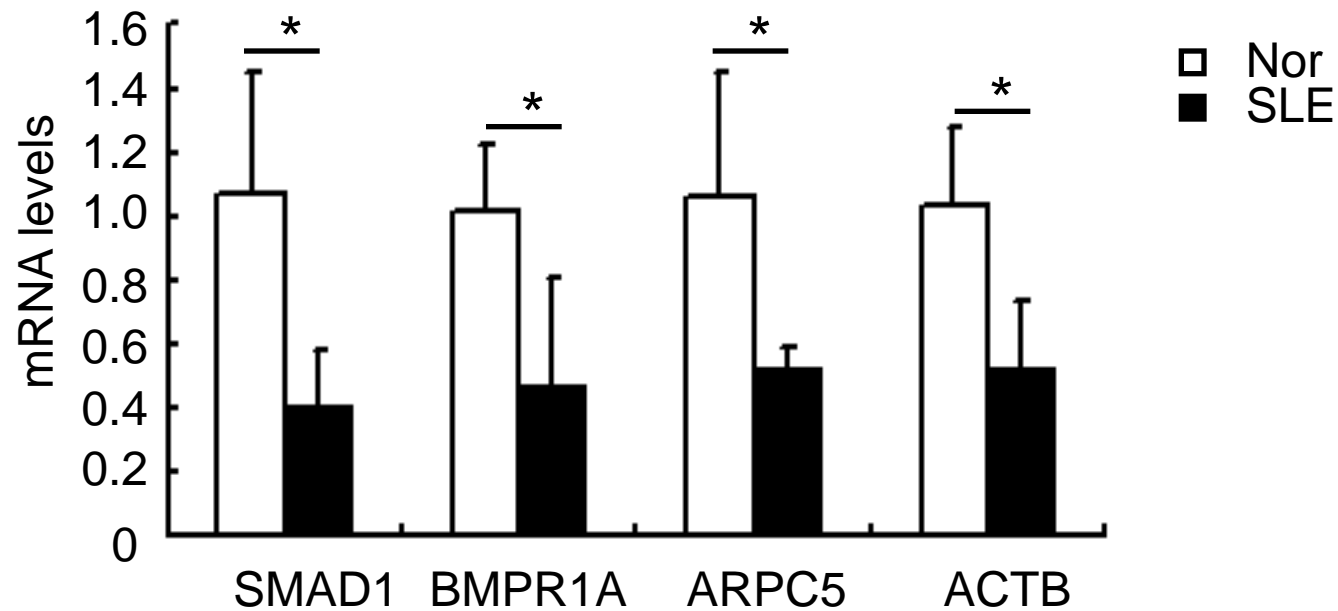

B

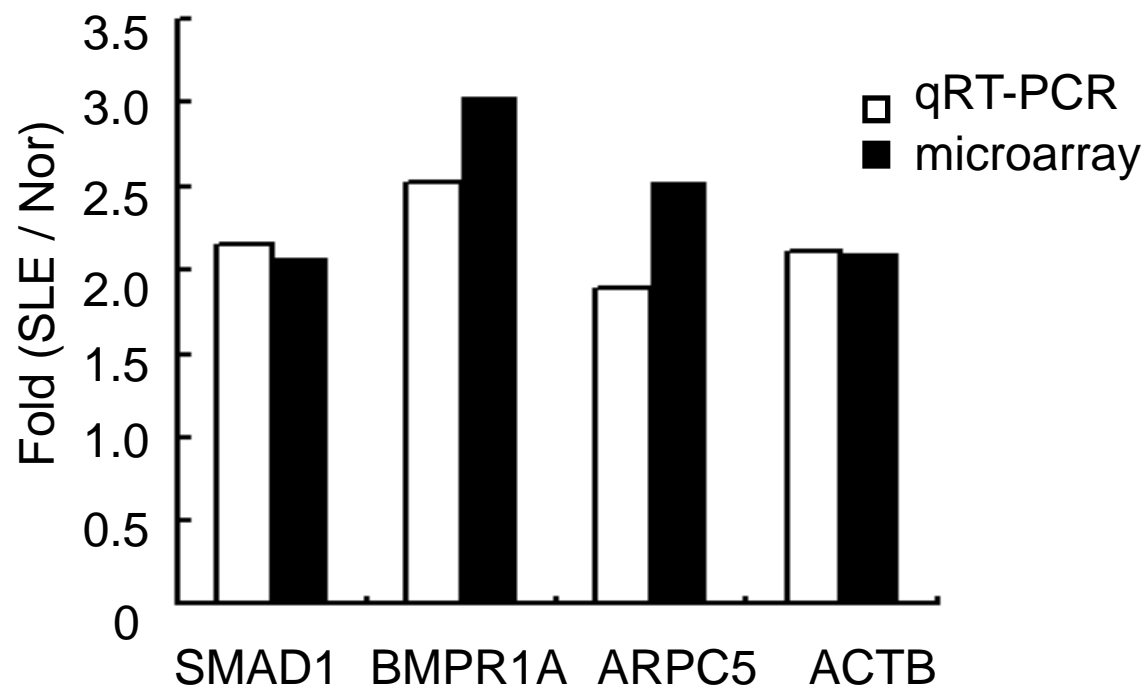

Supplement: Supplementary file 1 — Supplementary Figure 1: Verification of data from microarray hybridization by quantitative Real-time PCR. (A) The mRNA levels of SMAD1, BMPR1A, ACTB and ARPC5 in BMMSCs from SLE patients and normal controls by qRT-PCR. Results were shown as mean ± SEM, each performed with triplicate samples. ∗P<0.05 by Student's t-test (n = 4). (B) The ratio of SLE to normal controls. The folds of SLE to normal controls of Smad1, BMPR1, ARPC5, ACTB were 2.16-, 2.52-, 1.89- and 2.12 respectively by qRT-PCR. The intensity folds of normal to SLE BMMSCs of selected genes between microarray and qRT-PCR were assessed using the independent Student's t-test. There was no statistical significance in the ratios between the methods of microarray and qRT-PCR (n = 4, P>0.05). SLE: systemic lupus erythematosus, Nor: normal controls. Supplementary Figure 2: Flow cytometry analysis showed that the BMMSCs from SLE patients at passage three were CD29, CD44 and CD105 positive, while CD14, CD34, CD45, and HLA-DR negative cells. The shadow showed negative controls. SLE: systemic lupus erythematosus, Nor: normal controls. Supplementary Figure 3: The correlation of Id1 with TNF-α in normal controls. Id-1 mRNA levels of BMMSCs from normal controls had no correlation with their serum levels of TNF-α (n = 10, p = 0.76). [file 826182.f1.pdf]

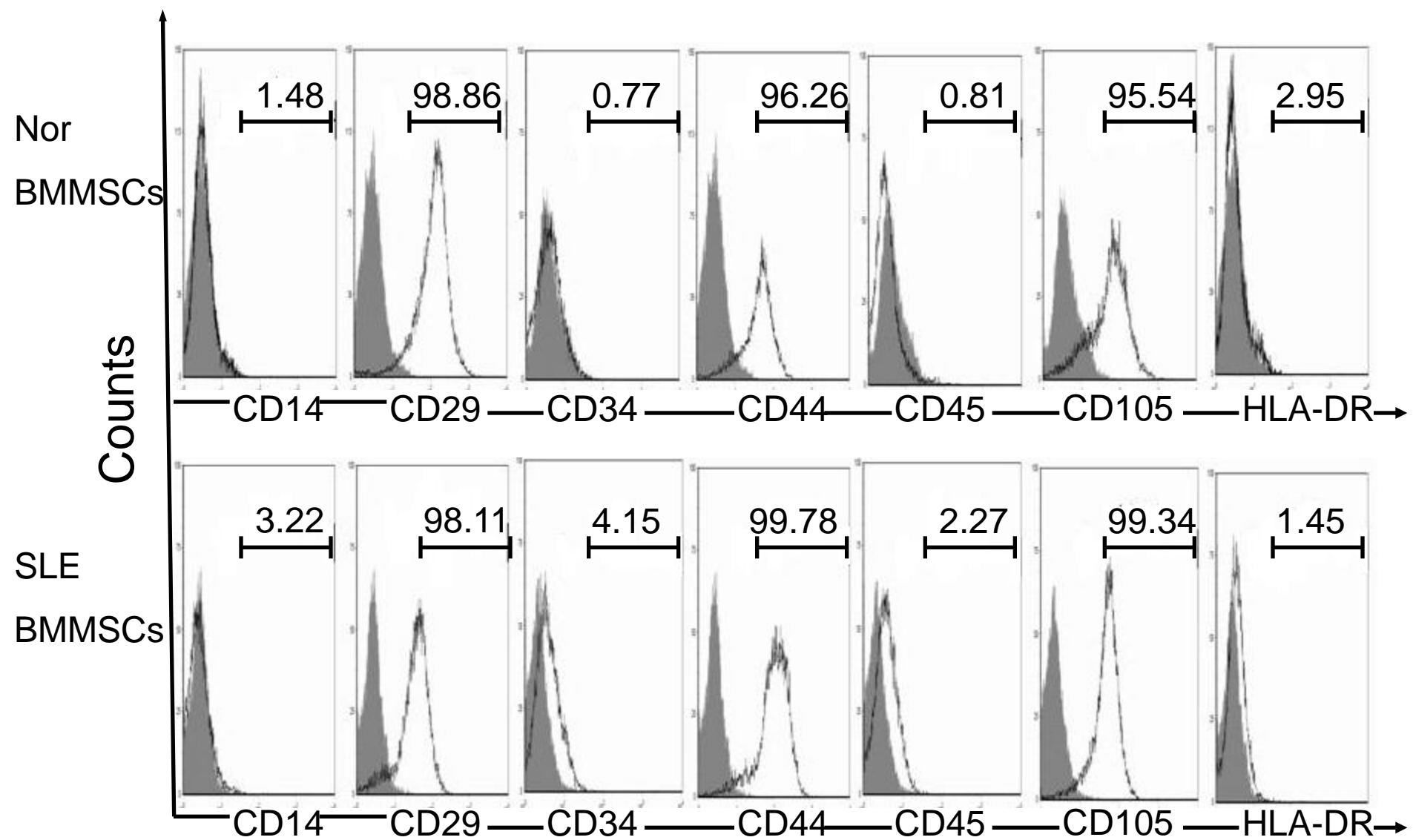

Supplement: Supplementary file 2 [file 826182.f2.pdf]

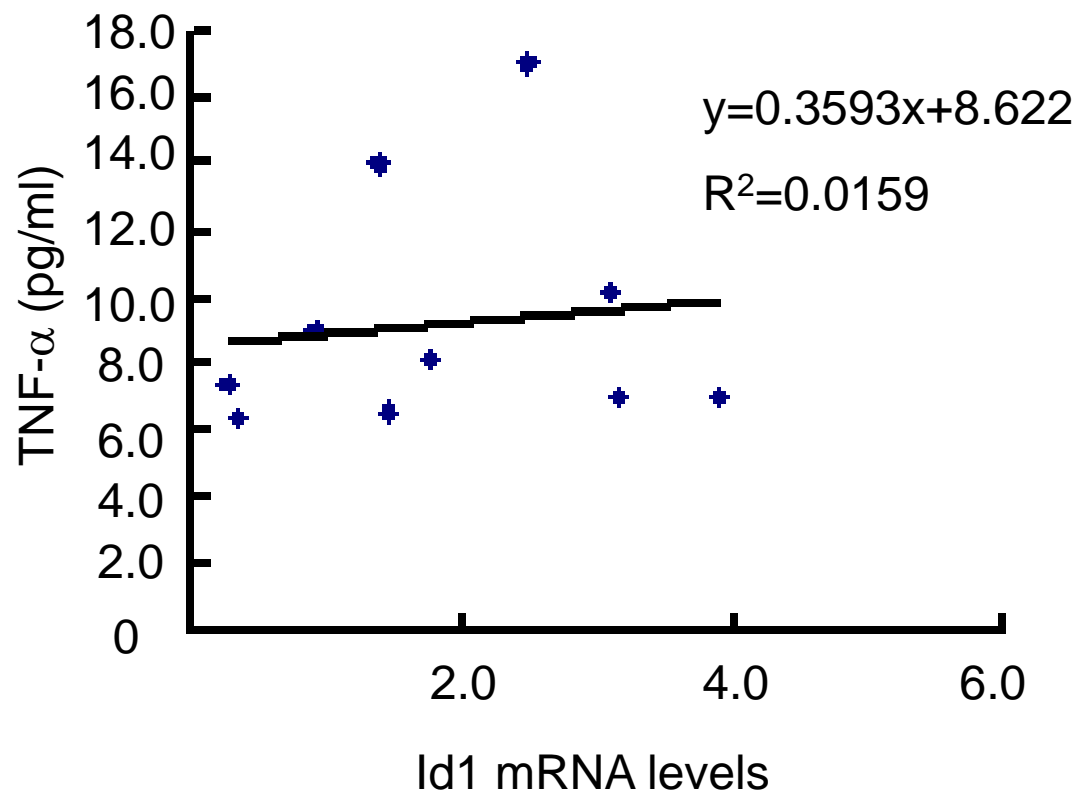

Supplement: Supplementary file 3 [file 826182.f3.pdf]
